# Inducible HSP70 Antagonizes IL-1β Cytocidal Effects through Inhibiting NF-kB Activation via Destabilizing TAK1 in HeLa Cells

**DOI:** 10.1371/journal.pone.0050059

**Published:** 2012-11-21

**Authors:** Xiang Cao, Ling Yue, JiYun Song, Qiuyue Wu, Na Li, Lan Luo, Lei Lan, Zhimin Yin

**Affiliations:** 1 Jiangsu Province Key Laboratory for Molecular and Medical Biotechnology, College of Life Science, Nanjing Normal University, Nanjing, Jiangsu, People’s Republic of China; 2 State Key Laboratory of Pharmaceutical Biotechnology, School of Life Sciences, Nanjing University, Nanjing, Jiangsu, People’s Republic of China; Technische Universitaet Muenchen, Germany

## Abstract

**Background:**

Despite several reports describing the HSP70-mediated cytoprotection against IL-1, the precise mechanism for this phenomenon remains to be determined.

**Methods/Principal Findings:**

Here we used HeLa cells, a human epithelial carcinoma cell line, to evaluate the role of inducible HSP70 in response of IL-1β stimulation. We found that inducible HSP70 antagonized the cytotoxicity of IL-1β and improved the survival of HeLa cells. Further investigation demonstrated that increased expression level of inducible HSP70 reduced the complex of TAK1 and HSP90, and promoted the degradation of TAK1 protein via proteasome pathway. By overexpression and RNAi knockdown, we showed that inducible HSP70 modulated the NF-kB but not MAPKs signalings through influencing the stability of TAK1 protein in HeLa cells. Moreover, overexpression of HSP70 attenuated the production of iNOS upon IL-1β stimulation, validating that inducible HSP70 serves as a cytopretective factor to antagonize the cytocidal effects of IL-1β in HeLa cells.

**Conclusions/Significance:**

Our observations provide evidence for a novel signaling mechanism involving HSP70, TAK1, and NF-κB in the response of IL-1β cytocidal effects. This research also provides insight into mechanisms by which HSP70 exerts its cytoprotective action upon toxic stimuli in tumor cells.

## Introduction

Interleukin-1 (IL-1) family is a pleiotropic cytokine produced mainly by activated monocytes/macrophages, but also expressed in a variety of other immune and non-immune cells. IL-1 plays a major role in the regulation of immune and inflammatory responses resulting in not only fever, fatigue, arthritis, and other constitutional symptoms but also tissue damage and infections [1], [2]. It has been demonstrated that IL-1 exerts a wide range of activities, including the regulation of growth, differentiation and many metabolic processes in a variety of cell types [3], [4]. The role and mechanism of IL-1 in mediating inflammation have been extensively studied. Two major forms of IL-1, IL-1α and IL-1β, although they have only 26% homology at the amino acid level, display similar characters in variety of biological functions both in vitro and in vivo [3]. IL-1α or IL-1β binds first to the ligand-binding chain, termed the type I IL-1 receptor (IL-1RI). This is followed by recruitment of the coreceptor chain, termed the receptor accessory protein (IL-1RAcP). The intracellular signal transduction is initiated with recruitment of the adaptor proteins MyD88 and Toll-IL-1 receptor domain followed by recruiting IL-1 receptor-associated kinase (IRAK) to this complex [5]. IRAK is phosphorylated at the receptor complexes and then in turn brings tumor necrosis factor-α receptor-associated factor 6 (TRAF6) to transforming growth factor β activated kinase (TAK1). Activated TAK1 subsequently triggers a number of downstream signaling cascades, including NF-κB, p38-MAPK, and JNK, leading to the activation of transcription factors such as NF-κB and AP-1 [6], [7].

Heat shock proteins (HSPs) are a group of phylogenetically conserved proteins found in all prokaryotic and eukaryotic cells and are categorized into five major families named on the basis of their approximate molecular weight [8]. The most studied and highly conserved HSPs is the HSP70 family, including both constitutively expressed and stress-inducible members. HSP70 proteins function as ATP-dependent molecular chaperones that assist in folding of newly synthesized polypeptides, the assembly of multiprotein complexes, transport of proteins across cellular membranes, and targeting of proteins for lysosomal degradation [9], [10]. Inducible HSP70 accelerates the cellular recovery by enhancing the ability of stressed cells to cope with increased concentrations of unfolded/denatured proteins upon many different types of stresses [11], [12] and they are also known to contribute to the mechanisms of cell protection against a variety of human diseases and cytotoxic factors [13], [14].

Besides its pro-inflammatory activity, IL-1 is able to exert cytostatic and cytotoxic effects on both transformed cells and tumor cells [15], [16], [17], [18], [19]. To date, the cytocidal action of IL-1 has been associated with the induced apoptosis via activation of NF-κB, inducible nitric oxide synthase (iNOS), and Bax protein [20] and the induced production of NO [17], [21]. It has been reported that overexpression of HSP70 protects rat pancreatic islet β-cells against IL-1β induced impairments [22] and also protects rat glomerular mesangial cells against IL-1β induced apoptosis [23]. However, the detailed signaling mechanism underlying HSP70 cytoprotective function upon IL-1β is largely unknown. *Hsp70* knockout mice revealed that the absence of *Hsp70* can significantly increase the activation of NF-κB, and the inflammatory cytokine response [24]. In addition, our previous study showed that HSP70 inhibits lipopolysaccharide (LPS)-stimulated NF-κB activation by interacting with TRAF6 and decreases the production of inflammation mediators such as iNOS and cyclooxygenase-2 [25].

Doszczak et al. recently showed that IL-1 exerts cytocidal effect on HeLa cells only in the presence of cycloheximide (CHX), a protein synthesis inhibitor [19], however the reason for this phenomenon is unclear. In this report, we analyzed the role of inducible HSP70 in the antagonism of IL-1 cytotoxicity in HeLa cells. We found that overexpression of inducible HSP70 prevented IL-1β-induced cytotoxicity in HeLa cells exposed to both IL-1β and CHX. Then we assessed the behavior of TAK1, an upstream activator of NF-κB signaling, in mediating NF-κB activation. We demonstrated that overexpression of inducible HSP70 reduced the complex of TAK1 and HSP90, the formation of which has been previously shown essentially to keep TAK1 stability in murine macrophages [26], and promoted the degradation of TAK1 protein via proteasome pathway, thereby inhibiting NF-kB activation and resulting in an increase of cell viability of HeLa cells upon IL-1β stimulation. We provide evidence for a novel signaling mechanism involving HSP70, TAK1, and NF-κB in the response of IL-1β cytocidal effects.

## Materials and Methods

### Antibodies and Reagents

Monoclonal antibody against HSP90 and Rabbit polyclonal antibody against HSP27 were obtained from Stressgen Bioreagents. Antibodies against IκBα(C-21), goat polyclonal antibody against HSP70 and Protein A/G, were from Santa Cruz Biotechnology. Polyclonal antibody against TAK1 was from Upstate. Polyclonal antibodies against JNK/SAPK, phospho-JNK/SAPK (Thr183/Tyr185), p38-MAPK, phospho-p38-MAPK (Thr180/Tyr182) and β-actin were obtained from Cell Signaling Technology. Mouse monoclonal antibody against GAPDH was purchased from Roche Applied Science. Monoclonal antibodiy against iNOS was from Biosciences Pharmingen. Secondary antibodies coupled to IRDye800 flurophore for use with the Odyssey Infrared Imaging System were purchased from Rockland. Recombinant human IL-1β was purchased from Chemicon. Cycloheximide (CHX) was obtained from Calbiochem. Monoclonal antibody against Flag-tag and Arbobenzoxylleucinyl-leucinyl-leucinal-H (MG-132) were from Sigma. Dimethyl sulfoxide (DMSO) was from Amresco.

### DNA Constructs

The pcDNA3.0-Flag-HSP70 was kindly provided by Dr. Chen Wang (Chinese Academy of Sciences, Shanghai, P.R. China). Small hairpin RNA (shRNA) against inducible HSP70 was constructed into pRNA-U6.1/neo and used as previously described [27]. The constructs were verified by DNA sequencing and purified using the Endofree Plasmid Preparation Kit (Qiagen).

### Cell Culture and Transfection

Cervical carcinoma HeLa cells were maintained in Dulbecco’s modified Eagle’s medium (HyClone) containing 10% (v/v) fetal bovine serum (HyClone) and antibiotics (100 U/ml penicillin and 100 µg/ml streptomycin) at 37°C in an atmosphere of 5% CO2. For transient transfection, HeLa cells were plated at a density of 5×10^4^ cells per well in six-well plates. At the following day, cells were transfected with 2 µg of the expression constructs with the use of the LipofectAMINE 2000 reagent (Invitrogen) or FuGene6 (Roche Applied Science, Basel, Switzerland) according to the manufacturer’s instructions. In all cases, the total amount of DNA was normalized by the empty control plasmids.

### Cell Viability/toxicity Assay

HeLa cells were seeded into 96-well plates at a density of 5×10^3^ cells per well for 24 h before treatment. After indicated treatments, cell viability was determined using Cell Counting Kit-8/CCK-8 (Beyotime) according to the manufacturer’s instruction. Briefly, 10 µL of CCK-8 working solution was added into each well and incubated at 37°C for 1–4 h. The absorbance of each well at 450 nm was measured using a Synergy2 Multi-Mode Microplate Reader (BIO-TEK, INC). Three replicates were carried out for each of the different treatments.

### Co-immunoprecipatations and Immunoblotting Analysis

HeLa Cells were lysed on ice in the lysis buffer (20 mM Tris pH 7.5, 135 mM NaCl, 2 mM EDTA, 2 mM DTT, 25 mM β-glycerophosphate, 2 mM sodium pyrophosphate, 10% glycerol, 1% Triton X-100, 1 mM sodium orthovanadate, 10 µg/ml aprotinin, 10 µg/ml leupeptin, and 1 mM phenylmethylsulfonyl fluoride) supplemented with complete protease inhibitor cocktail (Roche Applied Science, Indianapolis, IN, USA). Lysates were centrifuged (12,500×g) for 15 min. Proteins were immunoprecipitated with indicated antibodies separately at 4°C for overnight. The precleared Protein A/G Plus-Agarose beads (Santa Cruz Biotechnology) were incubated with immunocomplexes for 3 h and washed four to five times with the lysis buffer. The immunoprecipitates were subjected to SDS-PAGE followed by transferring onto nitrocellulose membranes (Whatman, GE Healthcare, NJ, USA). The antibody-antigen complexes were visualized by the LI-COR Odyssey Infrared Imaging System according to the manufacturer’s instruction using IRDye800 flurophore-conjugated antibody (LI-COR Biosciences, Lincoln, NE). Quantification was directly performed on the blot using the LI-COR Odyssey Analysis Software. Aliquots of whole cell lysates were subjected to immunoblotting to confirm appropriate expression of proteins.

### RT-PCR Analysis

Total RNA was extracted from the cells using TRIzol reagent (Invitrogen) and treated with RQ1 DNase (Promega). The reverse transcription (RT) and PCR amplification were performed using the Access RT-PCR Introductory System with indicated primer pairs according to the manufacturer’s instruction (Promega). Primer sequences are as follows:

TAK1 forward, 5′-TGGACGTTTAAGCTTGGGAGC-3′;

TAK1 reverse, 5′-CCAGTTCTGCAACTAGTTCTTGC-3′;

β-actin forward, 5′-TCATGAGGTAGTCAGTCAGG-3′;

β-actin reverse, 5′-TGACCCAGATCATGTTTGAG-3′.

Briefly, RT was performed by incubation of 25 µl of reaction mixture at 45°C for 45 min, and PCR was subsequently performed for 30 cycles at 94°C (30 seconds), 56°C (30 seconds), and 68°C (30 seconds) followed by a final extention at 68°C for 10 min. PCR products were seperated on a 1.2% agarose gel and stained with ethidium bromide. β-actin was used as an internal control.

### RNA Interference

HeLa cells were transfected with pRNA-U6.1/neo-HSP70-RNAi or empty vector (pRNA-U6.1/neo) with FuGene6 (Roche Applied Science, Basel, Switzerland) according to the manufacturer's instructions. After 48 h transfection, cells were stimulated with IL-1β (10 ng/ml) for indicated times and subjected to immunoblot analysis using indicated antibodies. Interference efficiency was evaluated by immunoblot analysis. Briefly, HeLa cells were transfected with pRNA-U6.1/neo or pRNA-U6.1/neo-HSP70-RNAi and after 48 h transfection, cells were exposed to IL-1β (10 ng/ml) for 12 h and cell lysates were subjected to immunoblotting with HSP70 antibody.

### Statistical Analysis

Data were represented as mean ± SD. We performed statistical comparison by Student’s *t*-test. A value of *P*<0.05 was considered statistically significant. Statistical calculations were performed by SPSS 13.0 software.

## Results

### HSP70 Overexpression Antagonized IL-1β-induced Cytotoxic Effect on HeLa Cells

It has been known for a long time that IL-1 is a cytocidal factor for several tumor cell lines except for human epithelial carcinoma cell line, HeLa [15]. Recently, Doszczak et al. reported that IL-1 exerts cytocidal effects only in the presence of protein synthesis inhibitor CHX, but the reason for this phenomenon is unclear [19]. This observation led us to hypothesize that the synthesis of certain proteins whose expression could antagonize IL-1-induced cytotoxic effect was suppressed by CHX. One likely candidate is HSP70, a well known cytoprotective factor. We thus initiated our research to investigate the correlation of stess-inducible HSP70 with IL-1 cytotoxicity in HeLa cells.

**Figure 1:**
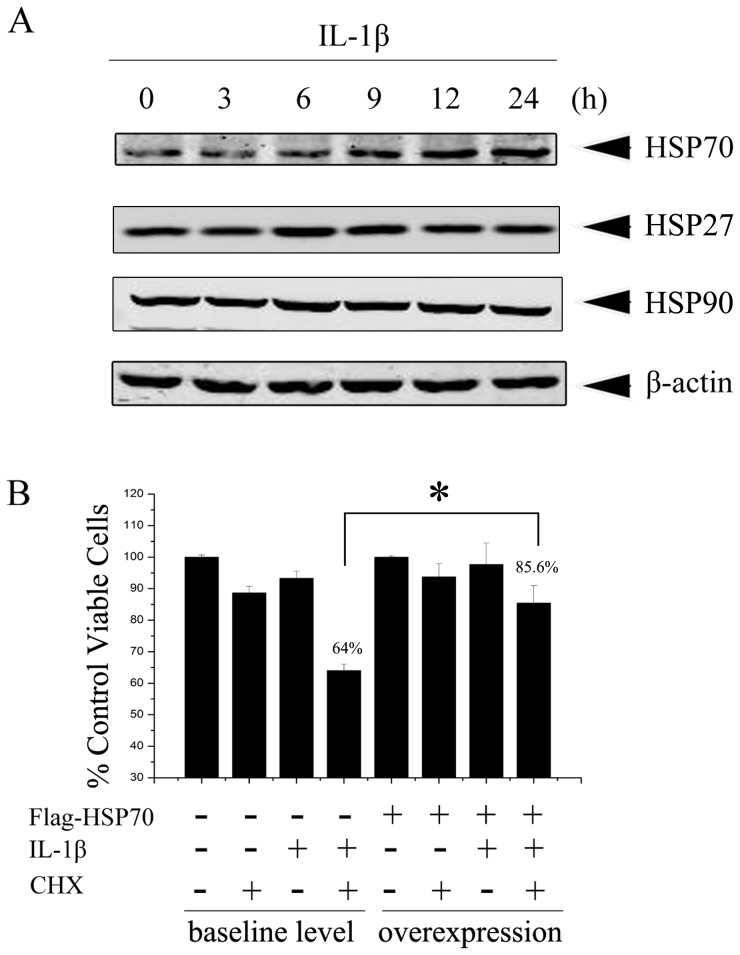
Inducible HSP70 antagonized IL-1β-induced cytotoxic effect on HeLa cells and improved the cell survival. (A) HeLa cells were stimulated with IL-1β (10 ng/ml) for indicated times, HSP70, HSP27, HSP90, and β-actin levels were determined by Western blotting. (B) HeLa cells were transfected with pcDNA3.0 (−) or pcDNA3.0-Flag-HSP70 (+). After 48 h, cells with either baseline level of or overexpression of HSP70 were exposed to IL-1β(10 ng/ml) or CHX (1 µg/ml) or both for 24 h. Cell viability was subsequently detected by CCK8 assay as described in Material and Methods. Cell viability is shown relative to the untreated control. The experiment was independently repeated for three times and data were shown as mean ± SD; bars: SD. Significant difference was determined by student’s *t*-test comparing HSP70 transfected cells with vector transfected control exposed to both IL-1β and CHX, * *P*<0.05.

Upon IL-1β stimulation, the expression level of HSP70 was significantly increased along with time compared to the constant expression of β-actin, however the expression of other important HSPs, HSP90 or HSP27 was not changed (Figure 1A). Next, we performed cell viability assay to compare the IL-1β-induced cell death in HeLa cells with or without HSP70 overexpression in the presence or absence of CHX. As shown in figure 1B, IL-1β treatment alone did not cause obvious cell death in HeLa cells, while combined treatment of IL-1β and CHX caused up to 36% of HeLa cell death. By contrast, only less than 15% of cell death was observed in group transiently overexpressed with HSP70 exposed to both IL-1β and CHX, demonstrating that inducible HSP70 protects HeLa cells from IL-1β cytotoxicity.

### Inducible HSP70 Down-regulated Endogenous TAK1

**Figure 2:**
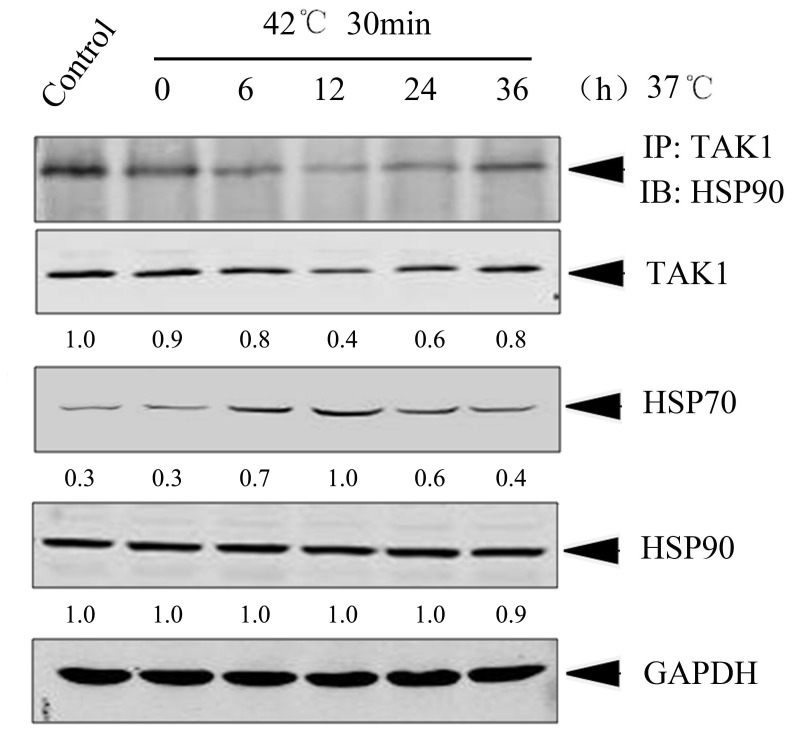
Increased expression level of HSP70 upon heat shock reduced TAK1 protein level and the association between HSP90 and TAK1. HeLa cells were either untreated (control) or exposed to a mild heat shock (42°C for 30 min) followed by recovery at 37°C for indicated time periods. Cell lysates were prepared and subjected to immunoblotting with indicated antibodies or immunoprecipitation with TAK1 antibody. The immunopellets were analyzed by immunoblotting with HSP90 antibody. Equal loading protein was confirmed by GAPDH. The numbers below each Western blot represent relative expression level normalized to GAPDH, which were determined based on the intensity of band. These experiments were independently repeated for three times, and the representative blots were shown.

It has been previously demonstrated that TAK1 is an essential mediator of IL-1 signaling [28], [29]. We therefore intended to investigate the effect of inducible HSP70 on the TAK1. Heat shock was employed to physiologically enhance the expression level of inducible HSP70. HeLa cells were incubated at 42°C for 30 min followed by recovery at 37°C for indicated time periods. Immunoblotting revealed that the protein level of inducible HSP70 was obviously increased upon heat shock (Figure 2), while the expression of HSP90, another important member of HSPs, was not obviously elevated (Figure 2). The level of inducible HSP70 reached to its maximum at 12 h after 37°C recovery, and then attenuated to its baseline level from 36 h onward. Interestingly, we found that the expression of TAK1 was decreased along with the increase of HSP70 and *vise versa* (Figure 2). It has been previously reported that HSP90 regulates IL-1β-induced signaling by interacting with TAK1 [26] and HSP90 is required for the folding and stability of TAK1 [30]. We thus further determined the impact of inducible HSP70 on the complex of HSP90-TAK1 in HeLa cells. Co-immunoprecipitation clearly showed that the endogenous TAK1 binds to HSP90 and the association of HSP90 and TAK1 was negatively correlated with the protein level of inducible HSP70 (Figure 2). These observations indicate that inducible HSP70 may affect the complex formation between HSP90 and TAK1 and destabilize TAK1 protein.

### HSP70 Destabilized TAK1 at Protein Level via Proteasome Pathway

**Figure 3:**
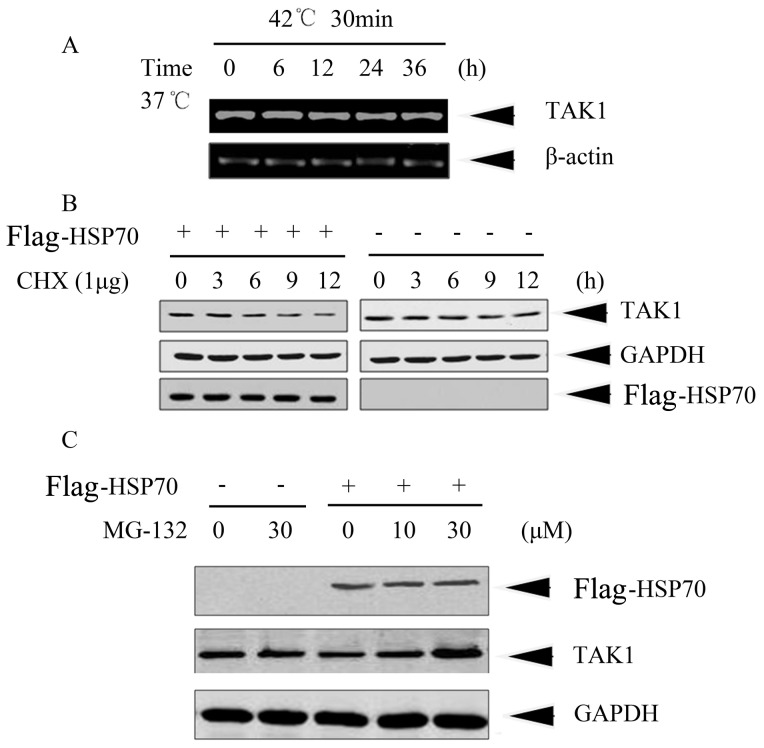
HSP70 destabilized TAK1 at protein level via proteasome pathway. (A) HeLa cells were exposed to a mild heat shock (42°C for 30 min) followed by recovery at 37°C for indicated time periods. Total RNA was isolated and TAK1 mRNA was detected by RT-PCR using primers indicated in Material and Methods. β-actin was used as control. (B) HeLa cells were transfected with pcDNA3.0-Flag-HSP70 (+) or pcDNA3.0 (−). After 48 h, cells were treated with 1 µg/ml cycloheximide (CHX) and harvested at indicated time points. TAK1 protein levels were determined by Western blotting, GAPDH was used as loading control, and antibody against Flag-tag was used to show the overexpression of HSP70. (C) HeLa cells were transfected with pcDNA3.0-Flag-HSP70 (+) or pcDNA3.0 (−). After 48 h, cells were treated with a proteasome inhibitor MG-132 at indicated concentrations for 6 h. Cells were collected and subjected to immunoblot analysis with TAK1 antibody. Equal loading protein was confirmed by immunoblotting with anti-GAPDH antibody.

To further investigate that HSP70 influences TAK1 stability, we first checked the change of TAK1 at transcriptional level using RT-PCR. HeLa cells were incubated at 42°C for 30 min followed by recovery at 37°C for indicated time periods. Total RNA was extracted and RT-PCR was conducted to reveal mRNA level of TAK1. The result showed that heat shock and recovery treatments did not influence the endogenous TAK1 at mRNA level, suggesting that inducible HSP70 affects the TAK1 at protein level (Figure 3A). Subsequently, to strictly exclude the effects of other major HSPs, we conducted HSP70 overexpression by transient transfection HeLa cells with pcDNA3.0-Flag-HSP70 to directly determine the effects of inducible HSP70 on TAK1 protein and on IL-1β signaling in the following experiments. After 48 h of transfection, CHX, a protein synthesis inhibitor, was added into culture medium for indicated times and cell lysates were subjected to immunoblotting. As shown in Figure 3B, in the presence of CHX, TAK1 protein level was significantly decreased in HSP70-overexpressing HeLa cells but not in vector-transfection HeLa cells, demonstrating that inducible HSP70 affected the stability of TAK1 protein. To further determine how HSP70 down-regulates TAK1, we employed proteasome inhibitor MG132 to terminate the protein degradation through proteasome pathway. After 48 h of transfection with pcDNA3.0-Flag-HSP70, HeLa cells were treated with MG132 at different doses (0, 10 µM, 30 µM) for 6 h and TAK1 was determined by immunoblotting. Figure 3C clearly showed that MG132 apparently caused an accumulation of TAK1 in HSP70-overexpressing HeLa cells compared with the empty vector transfected cells, indicating that inducible HSP70 down-regulated TAK1 protein through a proteasome-dependent pathway.

**Figure 4:**
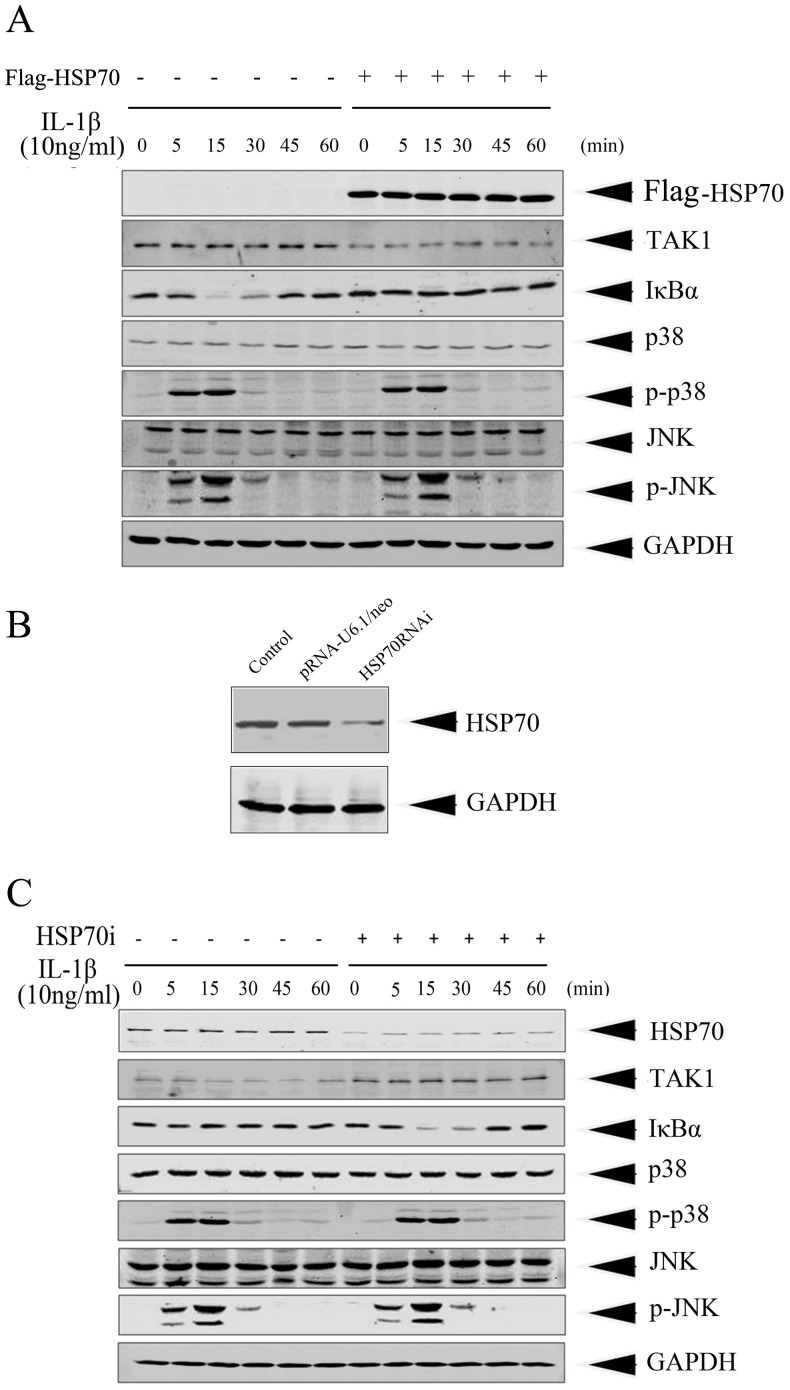
Inducible HSP70 modulated IL-1β-induced activation of TAK1-NF-κB cascades, but not of TAK1-MAPKs. (A) HeLa cells were transfected with pcDNA3.0 (−) or pcDNA3.0-Flag-HSP70 (+) for 48 h and then stimulated with IL-1β (10 ng/ml) for indicated times. Cell lysates were prepared and subjected to immunoblotting with anti-IκBα, anti-phospho-p38 (p-p38, Thr180/Tyr182), and anti-phospho-JNK (p-JNK, Thr183/Tyr185) antibodies, respectively, to reveal the activation of NF-κB and MAPKs. Total cellular TAK1, p38, and JNK protein levels in cell lysates were also determined by immunoblotting with specific antibody respectively. (B) HeLa cells were either non-transfected (control) or transfected with pRNA-U6.1/neo vector or pRNA-U6.1/neo-HSP70i construct. After 48 h transfection, cells were exposed to IL-1β (10 ng/ml) for 12 h and cell lysates were subjected to immunoblotting with anti-HSP70 antibody to determine the efficiency of RNAi, GAPDH was used as an internal control. (C) HeLa cells were transfected with either pRNA-U6.1/neo vector or pRNA-U6.1/neo-HSP70i construct. After 48 h transfection, cells were stimulated with IL-1β (10 ng/ml) for indicated times. immunoblotting with anti-IκBα, anti-phospho-p38 (p-p38, Thr180/Tyr182), and anti-phospho-JNK (p-JNK, Thr183/Tyr185) antibodies, respectively, to reveal the activation of NF-κB and MAPKs. Total cellular TAK1, p38, and JNK protein levels in cell lysates were also determined by immunoblotting with specific antibody respectively. Equal loading protein was confirmed by GAPDH.

### HSP70 Modulated IL-1β-induced Activation of TAK1-NF-κB Cascades, but not of TAK1-MAPKs

As we showed above, inducible HSP70 antagonized IL-1β-induced cytotoxicity and destabilized TAK1, we therefore moved forward to investigate whether IL-1β-induced TAK1-mediated downstream pathways were affected. It has been shown that TAK1 is an MAPKKK in IL-1β-triggered MAPKs (JNK, p38) and NF-κB signaling pathways [6], [31], [32], [33]. Here, we studied whether HSP70 overexpression could inhibit NF-κB and MAPKs cascades via destabilizing TAK1 protein. HeLa cells were transfected with pcDNA3.0-Flag-HSP70 or with empty vector as control and after 48 h of transfection, cells were stimulated with 10 ng/ml of IL-1β for indicated times. The activation of NF-κB signaling was evaluated by detection of IκBα degradation, and the phosphorylations of p38 and JNK were considered as the activation of MAPKs signaling. By immunoblotting, as shown in Figure 4A, HSP70 overexpression evidently reduced the TAK1 protein level. The activation of NF-κB began from 15 min upon IL-1β stimulation in HeLa cells transfected with empty vector, while the protein level of IκBα did not obviously changed in HSP70-overexpressing HeLa cells, suggesting that HSP70 overexpression inhibited NF-κB activation probably via destabilizing TAK1. By contrast, the phosphorylation patterns of p38 and JNK upon IL-1β stimulation did not exhibit any obvious divergence between HeLa cells with or without HSP70 overexpression, indicating that inducible HSP70 did not affect TAK1-MAPKs pathway.

**Figure 5:**
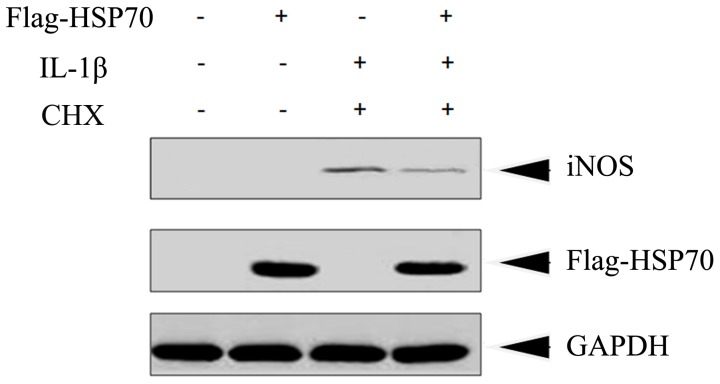
HSP70 overexpression inhibited IL-1β-induced production of iNOS. HeLa cells were transfected with pcDNA3.0 (−) or pcDNA3.0-Flag-HSP70 (+) for 48 h and then stimulated with or without IL-1β (10 ng/ml) plus CHX (1 µg/ml) for 4 h. Cells lysates were prepared and subjected to immunoblotting to measure protein level of iNOS. Antibody against Flag-tag was used to show the overexpression of HSP70 and equal loading protein was confirmed by GAPDH.

To further validate that inducible HSP70 inhibits NF-κB activation via destabilizing TAK1 upon IL-1β stimulation, we utilized specific small interference RNA (siRNA) against inducible HSP70 to knock down HSP70 and detected the correlation among HSP70 protein level, TAK1 protein level, and the NF-κB activation. HeLa cells were transfected with pRNA-U6.1/neo or pRNA-U6.1/neo-HSP70-RNAi and after 48 h transfection, cells were stimulated with IL-1β for 12 h and the expression of inducible HSP70 was examined by immunoblotting. By RNAi, inducible HSP70 can be reduced to about half of the level in control (without transfection) or empty vector transfected cells (Figure 4B). In HSP70 RNAi cells after IL-1β stimulation, TAK1 protein level was obviously higher than that in pRNA-U6.1/neo transfected cells. As expected, the activation of NF-κB occurred (as determined by IκBα degradation) only in the HSP70 RNAi but not in the vector transfected cells (Figure 4C). These observations further demonstrated that inducible HSP70 influenced the NF-κB activation by modulating TAK1 stability. Furthermore, knockdown of inducible HSP70 did not affect the activation of TAK1-MAPKs pathway (Figure 4C).

### HSP70 Overexpression Inhibited IL-1β-induced Production of iNOS

Besides the activation of NF-κB, iNOS also contributes to the cytotoxicity of IL-1 [20]. To further test the effect of HSP70 on IL-1 signaling, IL-1β-induced production of iNOS was also examined. HeLa cells were transfected with pcDNA3.0-Flag-HSP70 or with empty vector and after 48 h, cells were exposed to IL-1β (10 ng/ml) and CHX (1 µg/ml) for 4 h, and cell lysates were extracted and analyzed by Western blotting. As shown in Figure 5, HSP70 overexpression obviously attenuated the production of iNOS upon stimulation with IL-1β plus CHX, validating that inducible HSP70 serves as a cytoprotective factor to antagonize the cytocidal effects of IL-1β in HeLa cells.

## Discussion

HSP70 is induced in cells in response to a wide variety of chemical and physiological stresses and its expression provides protection against cell death. Unlike constitutively expressed heat shock cognate 70 (HSC70), inducible HSP70 is present at relative low level in untransformed cells, but is frequently observed at high level in tumor cells, in which HSP70 serves as a protective factor conferring resistance to stress-induced apoptosis [34] and suppression of default senescence pathway [35], and is associated with metastasis development and drug resistance [36]. IL-1 is a major pro-inflammatory cytokine that has several effects in the inflammation process. Growing evidence has also suggested that IL-1 exerts cytocidal effects on both primary cultured and transformed cell lines [19], [22], [23]. Several studies have examined the protective abilities of HSP70 against heat stress and toxic stimuli such as IL-1 [22], [23], however, the precise mechanism for this phenomenon remains to be determined.

In addition, Doszczak et al. recently reported that IL-1 exerts cytocidal effect on HeLa cells only in the presence of CHX, a protein synthesis inhibitor [19], but the reason for this phenomenon is unclear. We thus initiated our research to investigate whether inducible HSP70 was synthesized upon IL-1β stimulation in HeLa cells, which are human epithelial carcinoma cells, and we found an obvious increase of HSP70 level. Next, we determined the effects of HSP70 overexpression on the IL-1 cytotoxicity by cell viability assay. Consistent with the reported finding, IL-1β treatment alone did not cause obvious cell death unless combined with CHX. By contrast, HSP70 overexpression significantly improved cell survival under the treatment of IL-1β plus CHX, demonstrating that inducible HSP70 could prevent HeLa cells from cytocidal effects of IL-1β (Figure 1). As a further proof, we observed that HSP70 overexpression obviously attenuated the production of iNOS upon stimulation of IL-1β plus CHX (Figure 5).

Although a basal level of inducible HSP70 expression was observed in HeLa cells, IL-1β further improved the expression of inducible HSP70, but not the expression of other important HSPs, HSP90 or HSP27 (Figure 1A). This phenomenon was also observed in our heat shock experiments, in which cells were cultivated at 42°C for 30 min, only inducible HSP70 protein level exhibited an apparent enhancement in HeLa cells (Figure 2). It is not unexpected since HSP70 is known to be the most highly stress-inducible HSPs [37]. Taken together, our observations allow us to attribute the protective action against IL-1β cytotoxicity to the inducible HSP70 in HeLa cells. We reasoned that inducible HSP70 physiologically increased to respond to the IL-1β stimulation, and in turn, antagonized the cytocidal effects of IL-1β in HeLa cells, whilst the enhanced expression of inducible HSP70 upon IL-1β stimulation was attenuated when CHX was added together with IL-1β resulting in significantly decreased cell viability.

We previously demonstrated that HSP90 forms a complex with TAK1 and keeps its stability [26]. Here, we further found that, upon heat shock, both the association of HSP90/TAK1 and TAK1 protein level oscillated along with the change of HSP70 protein level (Figure 2). Since HSP90 protein level was relatively constant under our heat shock condition, we therefore speculated that the decrease of TAK1 protein resulted from the increase of HSP70 protein level. Further investigation demonstrated that overexpression of inducible HSP70 down-regulated TAK1 protein through a proteasome-dependent pathway (Figure 3). In addition, we also speculated that HSP70 could interfere the association between HSP90 and TAK1. In fact, we tried to detect the association of HSP70 with TAK1 by co-immunoprecipitation in HeLa cells treated with or without IL-1β. Unexpectedly, we did not succeed in capturing their complex. We reasoned that one possibility is that the association of HSP70 with TAK1 is either transient or very weak and their encounter could not be captured by our co-precipitation procedure. More accurate determining the timing of protein-protein encounter or employing other skills such as protein fixation in co-precipitation procedure to stabilize the transient encounter complexes may be helpful to evaluate whether the effect of inducible HSP70 on TAK1 stability is direct or mediated via other components.

Subsequently, we investigated whether the signaling pathways downstream of TAK1 could also be affected by HSP70 overexpression. Through both overexpression and RNAi knockdown experiments, we correlated the inducible HSP70 with the TAK1 protein level and the NF-κB signaling. Overexpression of HSP70 destabilized TAK1 protein resulting in the inhibition of NF-κB, while HSP70 RNAi upregulated TAK1 protein resulting in the activation of NF-κB (Figure 4A, C). Considering that a basal level of inducible HSP70 expression was observed in HeLa cells, it is possible that inducible HSP70 constitutively modulates the homeostasis of TAK1 in order to limit the activation and intensity of NF-κB signaling in HeLa cells under physiological conditions.

Moreover, our data showed that inducible HSP70 modulated the activation of TAK1-NF-κB, but not of TAK1-MAPKs (Figure 4A, C), which implied that IL-1β-activated MAPKs are mediated by TAK1-independent pathway. This observation is inconsistent with the general notion that TAK1 is an essential mediator of IL-1 signaling and acts as common upstream kinase to IL-1β-triggered MAPKs (JNK, p38) and NF-κB signaling pathways [6], [31], [32], [33]. The differential activation of NF-κB and MAPKs to the TAK1 stability may be explained by their different intracellular regulatory mechanisms in HeLa cells. It is reasonable since that in addition to TAK1, JNKs and p38 MAPK have also been reported to be activated by other MAPK kinase kinase, such as MEKK1 [38] and MEKK3 [39], in response to selected stimuli. This observation is also intriguing since the profound roles of MAPKs cascades either in cancer development or cancer suppression are poorly understood, although it has been characterized that activation of Ras-MAPK pathway promotes survival of neurons [40]. Therefore, the potential functional relevance of activation of JNKs and p38 MAPKs in the survival or apoptosis of HeLa cells upon IL-1β stimulation will be investigated in future.

In conclusion, IL-1β stimulation increased the inducible HSP70 expression, and in turn, inducible HSP70 promoted the degradation of TAK1 via proteasome-dependent pathway, and inhibited the activation of NF-κB signaling. Therefore, in the absence of CHX, enhanced expression of inducible HSP70 antagonized the IL-1β-induced cytocidal effects in HeLa cells and improved the cell survival. In addition, although the relationship between HSP70 and NF-κB signaling has been shown, especially in inflammatory response [25], [41], [42], the TAK1 has not been reported yet as a mediator between them. Here, we showed that inducible HSP70 modulated the activation of NF-κB signaling via destabilizing TAK1 protein. We thus provide evidence for a novel signaling mechanism involving HSP70, TAK1, and NF-κB in the response of IL-1 cytocidal effects. Our findings not only provide insight into mechanism by which HSP70 exerts its cytoprotective action, but also highlight that targeting HSP70 represents a powerful approach for sensitizing tumor cells to cytocidal therapy.
